# Supplementary material for: Are carbon emissions trading and green financial instruments synergistic? -Comprehensive quantitative research based on content analysis
Source: PLoS One. 2024 Mar 7;19(3):e0298601. doi: 10.1371/journal.pone.0298601 (PMC10919601; doi:10.1371/journal.pone.0298601)
Supplement: S1 Data — (ZIP) [file pone.0298601.s001.zip › Data/地级市Z_F工作报告.zip!/║╙─╧/┐¬╖Γ╩╨/└┤╫╘╣1⁄2╓┌║┼í╛┬φ┐╦╩2╛▌═°í┐_2011.docx]

各位代表：

现在，我代表市人民政府，向大会作政府工作报告，请予审议，并请各位政协委员和列席人员提出意见。

一、2010年及“十一五”期间全市经济社会发展回顾

2010年，市政府在省委、省政府和市委的正确领导下，深入贯彻落实科学发展观，团结带领全市人民，抢抓机遇、科学谋划，负重拼搏、扎实工作，圆满完成了市十三届人大二次会议确定的各项任务。主要做了以下工作：

（一）全力保持跨越式发展良好态势。积极应对复杂经济形势，继续把保持经济平稳较快发展、保持经济社会跨越式发展良好态势作为首要任务，大力实施“八大工程”和“八大行动计划”，经济社会发展在困难形势下取得了显著成绩。全市生产总值完成930.2亿元，增长12.6%，其中第一产业增加值219.3亿元，增长4.6%；第二产业增加值400.7亿元，增长14.9%；第三产业增加值310.2亿元，增长14.5%。财政一般预算收入37亿元，增长25.3%；其中市本级16亿元，增长27.7%。全部工业增加值368.3亿元，增长15.9%。城镇以上固定资产投资394.7亿元，增长25.1%。社会消费品零售总额364.6亿元，增长20.2%。城镇居民人均可支配收入13695元，增长11.2%；农民人均纯收入5390元，增长14.8%。经济发展速度连续三年超过全省平均水平，主要经济指标增幅继续位居全省前列，其中城镇以上固定资产投资、社会消费品零售总额和第三产业增加值增幅均居全省第1位，财政一般预算收入、市本级一般预算收入增幅均居全省第4位，规模以上工业增加值增幅居全省第5位。

（二）持续蓄积跨越式发展能量。坚持强基础、增后劲，突出抓好开封新区建设，着力加快产业集聚区发展，进一步提升投资和项目对经济发展的支撑能力，跨越式发展的基础更实、后劲更足。强力推进开封新区建设。加大基础设施投入力度，新区一期、二期工程18条道路全部建成通车，三期、四期基础设施和BT项目进展顺利；黑岗口调蓄水库及东京大桥、金耀大桥、晋安大桥开工建设；陇海铁路以南工业区基础设施建设正式启动；新区道路建设里程达85.3公里，发展框架不断拉大。扎实开展新区项目建设年活动，全年项目投资突破100亿元，增长87%。河南煤化集团大型空分设备、铁塔橡胶工业园和北京汇源集团无菌生产线等重点工业项目即将建成投产；奇瑞（河南）汽车制造基地项目实现微车下线；瑞创通用机械制造基地项目大型拖拉机批量生产。职教园区建设进展顺利，市旅游学校、省医药技校、省化工技校和省医药学校等4所学校首批入驻。迁村并居工程扎实推进，闫砦、毛砦社区建设项目进入验收竣工阶段；瞿家寨、野厂社区全面开工建设。开封经济开发区晋升为国家级经济技术开发区。着力加快产业集聚区发展。按照“四集一转”发展思路，大力实施“产业集聚区建设工程”，全市8个产业集聚区累计完成投资209亿元，其中基础设施投资28亿元；新开工项目54个，施工项目281个，产业支撑能力和集聚效应进一步显现。开封新区被评为河南省创新型产业集聚区，开封空分特色产业园被批准为国家级特色产业基地。努力增加固定资产投资。强力实施转型升级“3135”工程，实施了300个重大建设项目，完成投资318.5亿元，带动项目总投资1078亿元。2010年，全社会固定资产投资完成506.6亿元，增长24.7%。

（三）积极转变经济发展方式。坚持把扩总量、调结构作为加快转变经济发展方式的重点，积极培育新的经济增长点，提升发展的全面性、协调性和可持续性。不断增强工业经济实力。大力实施“企业服务行动计划”，积极帮助企业解决资金、土地、环境等问题。全市金融机构新增贷款84.5亿元，较年初增长25.7%，高出全省平均增幅7.6个百分点。实施“重点企业培育工程”，全市规模以上工业企业达到1207家；规模以上工业增加值276.3亿元，增长21.6%。开封发电、开封空分、新大新等11家企业主营业务收入超10亿元，晋开集团实现生产经营总值超30亿元。河南新大新材料股份有限公司正式登陆创业板。大力发展第三产业。实施“文化改革发展试验区建设工程”，开封宋都古城文化产业园区荣膺国家级文化产业示范园区。扎实推进重大文化旅游项目建设，延庆观玉皇阁顶升工程顺利完工；繁塔·禹王台景区建设项目一期工程、城墙保护四期工程、大宋文化博物馆·开封城摞城新郑门遗址、刘青霞故居综合开发等一批重点文化旅游项目加快推进。成功举办了“我们的节日—中国（开封）2010清明文化节”、中国收藏文化（开封）论坛、第十届中国菊花展览会暨中国开封第28届菊花花会，开封被授予“中国菊花名城”称号。积极开展景区创A工作，清明上河园荣膺5A级旅游景区。全市旅游接待量和旅游综合收入分别达到3469万人次和128.4亿元，增长12.1%和18.8%。大力发展金融保险等现代服务业，成功引进浦发银行，市商业银行在郑州设立支行。持续推进科技创新。深入实施“自主创新体系建设工程”，中国农业大学开封实验站挂牌成立，天民种业、十一化建获批设立博士后科研工作站；河南新大新材料股份有限公司、中平能化集团开封炭素有限责任公司工程技术研究中心被认定为省级工程技术研究中心。大力实施质量兴市和品牌带动战略，尉氏县久龙橡塑有限公司“耐驰”商标被评为“中国驰名商标”，实现了我市中国驰名商标零的突破。加强节能减排和生态环境建设。实行节能减排“倒逼”机制，关闭淘汰落后产能企业31家，全市单位GDP能耗较2009年下降7.95%，化学需氧量和二氧化硫排放量分别削减7%和1.1%，顺利完成“十一五”节能减排目标。坚持节约集约用地，增强用地保障能力，全市耕地总量连续12年实现占补平衡，建设用地获批突破3万亩。深入推进林业生态市建设，完成造林14万亩。全市城市环境空气质量优良天数比例达到84%；饮用水源地水质达标率继续保持100%。

（四）着力统筹城乡发展。坚持城乡统筹，以城带乡，切实加强基础设施建设，着力推进城镇化和农业现代化，促进城乡统筹协调发展。进一步夯实“三农”基础。加大财政支农力度，落实粮食直补、农资综合直补、家电下乡、汽车（摩托车）下乡等各类惠农资金8.3亿元。狠抓农田水利基本建设，新增灌溉面积7.2万亩；大力推广农业机械化，农业综合机械化水平达到75.3%；实施“粮食稳产保收行动计划”，全市粮食总产达到255.6万吨，增长0.8%，连续七年创历史新高。深入推进农业结构调整，农产品优质率较上年提高2个百分点；全市标准化养殖小区达到610个，规模养殖场发展到5869个。大力发展农业产业化经营，全市新增省级农业产业化龙头企业5个，农民专业合作社发展到1341家。实施社会主义新农村“百村示范”工程，21个省级示范中心村全面启动。加大扶贫开发力度，完成脱贫人口5.2万人。加强城乡基础设施建设。完成了黄河大街北延、集英街北延、金耀路中段、金耀路穿城墙、东京大道东延工程和龙亭湖景区周边环境综合整治，对五福路、五一路、大庆路等道路进行了改造，城市综合承载能力进一步增强。郑开物流通道、郑民高速（一期）、连霍高速开封段拓宽扎实推进。郑开城际铁路建设全线开工，宋城路站至火车站延伸批复立项。郑徐客运专线前期准备工作进展顺利。加强农村基础设施建设，新改建农村公路424.1公里，解决27.2万农村居民安全饮水问题，新建沼气池1万座，新建和改造农家店480个，建成村邮站1032个，改建农村卫生厕所1.1万座，农村面貌不断改善。加快城镇化进程。进一步完善规划编制，完成有关专项规划80余项，《开封市城市总体规划（2008—2020）》上报部际联席会议审议，《开封市综合交通规划》顺利通过专家评审。加快老城区改造，组建了宋都古城建设投资有限公司，小李庄安置用房及老城区1号、2号地块开发全面启动；升级改造了包公湖大市场、梁苑市场等4个农贸市场和东司门、武夷路2个夜市。城市数字化管理系统建成运行，精细化管理水平明显提高，一大批涉及城市管理和市民生活的问题得到及时有效解决。深入开展“创三城”工作，集中开展违法建设整治专项活动，加强环境卫生管理，市容市貌进一步改观。以基础设施建设为突破口，稳步推进朱仙镇名镇开发。2010年全市城镇化率达到41.2%，比上年提高1.6个百分点。

（五）全面深化改革开放。坚定不移地推进改革开放，以改革破难题，以开放增活力，在重点领域和关键环节取得了新突破。深入推进企业改革。实施“国企改革攻坚行动计划”，开化集团、缝纫机总厂、麦芽厂、柴油机厂、毛条厂、针织厂破产清算工作进入收尾阶段；中州机械厂职工安置工作基本完成；第二铸造厂、第二玻璃厂、联合制革公司进入职工安置阶段；开封联合收割机厂破产重组接近尾声，企业改革实现大头落地。扎实推进重点领域改革。市县政府机构改革圆满完成。警务机制改革顺利实施。市直事业单位岗位设置管理工作基本完成。医药卫生体制改革加快推进，乡镇卫生院人员分流安置工作基本结束。旅游景区管理体制改革取得突破，组建了市文化旅游投资集团有限公司并正式运营。继续压缩和规范行政审批事项，强力推行涉企收费“一费制”和项目代理制。财税体制改革以及收入分配、资源价格、环保收费等领域改革均取得了新的进展。进一步扩大对外开放。深入实施“对外开放行动计划”和“双百工程”，组织参加了一系列重大招商引资活动，成功承办第五届豫商大会，2010年全市新签亿元以上项目123个，总投资649.2亿元；新开工亿元以上项目108个，总投资566.7亿元；实际利用外资12882万美元，增长71%；外贸出口18801万美元，增长16%；引进省外资金177.6亿元，增长26.2%。我市被评为全省对外开放先进市。

（六）加快推进社会发展和民生改善。实施“十五项民生工程”，筹集资金30多亿元，解决了一批人民群众最关心、最直接、最现实的利益问题。做好就业和再就业工作。全市城镇新增就业人员6.6万人，失业人员再就业2.7万人，帮助困难人员就业1.2万人，动态消除了“零就业家庭”。全年转移农村劳动力89万人，实现劳务收入90.5亿元。提高社会保障水平。城市低保标准由每人每月180元提高到220元，农村低保对象每人每月补助60元，比2009年提高10元；农村五保年分散供养标准提高到1200元、集中供养标准提高到2000元，基本实现了应保尽保；为16.3万名企业退休人员人均每月增加养老金150元。在通许县开展了新型农村社会养老保险试点工作，为6.9万名60岁以上自愿参保农村居民发放养老保险金4011.8万元。加强价格管理和调控，对低收入群体进行补贴，保障群众基本生活。实施保障性安居工程。全市新开工经济适用住房98.4万平方米，竣工39.8万平方米，提供经济适用住房房源4022套；开工建设廉租住房21.4万平方米；为1.7万户城市低收入住房困难家庭累计发放租赁补贴5016万元。加快发展教育体育事业。发放农村中小学公用经费2.5亿元，免教科书费6675.2万元，为农村家庭困难寄宿生提供生活费补助1861.8万元，城市中小学免杂费2950万元；落实资金3.5亿元，推进中小学校舍安全工程。开封新区横堤铺小学、金明二小主体工程完工，开封高中西校区开工建设。扎实推进职业教育攻坚计划，开封文化艺术职业学院申办成功；河南大学民生学院开工建设，开封大学北校区建设加快推进。成功举办2010中国郑开国际马拉松赛，积极参加省第十一届运动会和省第六届少数民族运动会，金牌总数分别位居全省第4位和第3位。大力发展卫生事业。新改造2所县级医院、4所乡镇中心卫生院，完成492个行政村卫生室建设；市中医院病房楼建成投用，中心医院选址和建筑设计工作基本完成。开展新农合门诊统筹试点，财政补助标准提高到120元。积极做好人口和计划生育工作，人口自然增长率控制在5.5‰以下。进一步加强社区建设。新改（扩）建社区办公场所15个、社区卫生服务中心5个；社区工作人员待遇提高到每月700—800元。维护社会和谐稳定。实施“安全发展和平安建设行动计划”，全面完成“五五”普法验收工作，狠抓社会治安综合治理，严厉打击各类违法犯罪，切实加强信访稳定工作，强化安全生产管理，社会大局保持和谐稳定。深入开展食品药品安全专项整治，我市被评为省食品安全优秀城市。切实加强民主政治建设和政府自身建设。自觉接受人大及其常委会监督，支持政协履行职能，认真办理人大代表建议和政协提案。加强政府廉政建设，强化财政监察审计监督，反腐倡廉工作取得新成效。加强精神文明建设，公民道德水平和文明程度不断提升。大力开展国防教育，扎实做好“双拥”工作，驻汴解放军和武警部队在支持地方经济建设、维护社会稳定等方面作出了重要贡献。第六次人口普查工作进展顺利。对口援建江油任务全面完成。同时，民族宗教、社会科学、新闻出版、外事、侨务、对台、档案、史志、人防、地震、气象等工作都取得了新的成绩。

各位代表!

2010年各项工作目标的实现，为我市“十一五”发展画上了圆满句号。过去的五年，全市经济建设、政治建设、文化建设、社会建设以及生态文明建设都取得了崭新成就，谱写了建设富裕民主文明和谐新开封的美好篇章。

——过去的五年，是我市综合实力显著提升、经济实现跨越发展的五年。国民经济持续保持两位数增长，生产总值由“十五”末的408亿元增加到930亿元。全部工业增加值由不足150亿元增加到368亿元。财政一般预算收入由不足14亿元增加到37亿元。金融机构各项存款余额由315亿元增加到近700亿元。均比“十五”末翻了一番多。

——过去的五年，是我市经济结构明显优化、产业层次大幅提升的五年。全市三次产业比例由“十五”末的29.7∶40.2∶30.1调整到23.6∶43.1∶33.3，二、三产业占生产总值的比重上升6.1个百分点。农业农村经济稳步发展，粮食总产实现五连增。工业发展落后局面得到根本扭转，企业境内外上市实现零的突破。现代服务业快速发展，第三产业增幅始终位居全省前列。

——过去的五年，是我市基础设施建设不断加强、城乡面貌发生巨大变化的五年。累计投资近60亿元，建成城区道路145公里；对全市主次干道进行了改造整治，城区背街小巷基本改造完毕，主次干道无路灯现象基本消除。城市建设日新月异，开封新区建设全面展开，新农村建设扎实推进。城市建成区面积增加19平方公里，全市城镇化率提高8.5个百分点。

——过去的五年，是我市各项改革不断深化、对外开放取得重大突破的五年。企业改革大头落地；政府机构改革、事业单位改革以及财税管理、农村税费、警务机制等重大改革任务圆满完成。成功引进奇瑞汽车、中平能化、河南煤化、北大资源等一批战略投资者，累计引进国内500强企业15家，实际利用市外资金近千亿元，实施亿元以上重点项目322个，是引资规模最大、签约项目最多、合同金额最高、招商效果最好的五年。

——过去的五年，是我市民生明显改善、城乡居民得到更多实惠的五年。累计投入民生工程近百亿元。新增城镇就业再就业34.9万人。完成21.8万贫困人口脱贫。社会事业长足发展，公共服务体系不断完善，社会保障体系不断健全并逐步向农村延伸。城镇居民人均可支配收入、农民人均纯收入年均分别增长13.7%和14.7%，均高于“十五”平均水平。

各位代表！

五年的发展历程波澜壮阔，五年的辉煌成就令人自豪。这是省委、省政府和市委正确领导的结果，是市人大依法监督、市政协民主监督、社会各界大力支持的结果，是全市人民团结奋斗、顽强拼搏的结果。在此，我代表市人民政府，向全市广大工人、农民、知识分子和各级干部，向驻汴人民解放军指战员、武警官兵、公安民警，向关心和支持政府工作的各民主党派、工商联、各人民团体和社会各界人士，表示崇高的敬意和衷心的感谢！

各位代表！

回首“十一五”，我们坚持科学发展、跨越发展，历程极不平凡，经验弥足珍贵。通过实践，我们进一步深化了对科学发展观的认识，提高了贯彻落实科学发展观的能力。我们深刻体会到：第一，必须始终坚持实施“六大战略”，推进“三个跨越”。我们始终坚持以科学发展观为指导，把发展当作第一要务，唱响奋力实现经济跨越式发展的主旋律，坚定不移地实施工业强市、开放引进、旅游带动、科教兴汴、城市化和可持续发展战略，加快老工业基地向新型工业化城市跨越、传统农业向现代农业跨越、文化旅游大市向文化旅游强市跨越，实现了开封发展的重大转折，迈入了跨越式发展的新阶段。第二，必须始终坚持抢抓机遇，科学谋划。我们抢抓省委、省政府强力推进郑汴新区建设的重大机遇，积极谋划开封新区发展，重新确立了开封在全省的战略核心地位；抢抓开封被确定为比照实施振兴东北老工业基地政策的机遇，积极调整经济结构，工业经济迈上了发展的快车道；抢抓省委、省政府加快推进文化改革发展试验区建设的机遇，积极实施开封古城墙、水系二期等一批重大文化旅游产业项目，文化旅游产业发展提速增效；抢抓国际金融危机导致国际国内产业加快转移的机遇，积极承接产业转移，谋划和培育汽车及零部件等十大产业集群，为实现经济社会发展全面跨越奠定了坚实基础。第三，必须始终坚持解放思想，改革开放。我们始终坚持把解放思想作为应对新挑战、开创新局面的一大法宝，不断创新发展理念，转变发展方式；坚持用改革和发展的办法解决前进中的问题，强力推动改革向纵深推进、向广度拓展，广大干部群众的思想观念发生深刻变化，有效调动了各方面的积极性、创造性，进一步解放和发展了社会生产力。第四，必须始终坚持以人为本，改善民生。我们牢固树立让人民群众成为改革发展现实受益者的理念，把发展经济与改善民生相结合，大力实施民生工程，尽心尽力为群众办实事、办好事，解决民生问题的投入比以往任何时候都多，改善民生问题的成效比以往任何时候都好。第五，必须始终坚持统筹兼顾，协调推进。既立足当前抓项目、保态势，促进经济又好又快发展，又着眼长远抓工业、培三产，谋划新的经济增长点，为新一轮发展打基础、增后劲，努力实现经济社会全面协调可持续发展。这些经验和做法，是全市上下在改革发展稳定的伟大实践中不断创造和形成的，值得在今后的工作中继续坚持和发扬。

各位代表！

在肯定成绩的同时，我们也清醒地认识到，我市经济社会发展中还存在不少矛盾和问题，主要表现在：经济总量小、底子薄、县域经济发展滞后、经济外向度低的局面还没有得到根本改变；经济结构性矛盾比较突出，加快经济结构调整、转变经济发展方式的任务还十分繁重；城市基础设施欠账较多，农村公共服务和基础设施仍较薄弱，统筹城乡发展任重道远；影响改革、发展、稳定的难点和热点问题较多，一些涉及群众切身利益的问题还没有解决好，民生工作仍需继续努力；服务型政府建设还有一定差距，一些部门职能和作风转变还不到位，仍需继续加强政府自身建设，等等。对此，我们一定高度重视，认真解决。

二、“十二五”发展的总体要求和目标任务

“十二五”时期，是我市工业化、城镇化快速发展时期，是全面建设小康社会的关键时期，同时也是经济结构重大调整、产业升级加速推进、经济总量大幅提高、城市品位较大提升、居民收入较快增加、经济社会全面跨越发展的重要时期。

根据市委九届十二次全会精神，“十二五”全市经济社会发展的总体要求是：高举中国特色社会主义伟大旗帜，以邓小平理论和“三个代表”重要思想为指导，深入贯彻落实科学发展观，以科学发展为主题，以加快转变经济发展方式为主线，以把开封建设成为全省经济社会发展核心区为战略目标，牢牢把握重在持续、重在提升、重在统筹、重在为民的实践要领，抓住中原经济区上升为国家战略，省委、省政府持续推进郑汴一体化发展、把郑汴新区打造成为中原经济区核心增长极的重大机遇，坚持靠改革消除弊端、靠开放聚集资本、靠发展破解难题、靠实力赢得尊重，继续实施工业强市、开放引进、旅游带动、科教兴汴、城市化和可持续发展战略，着力转变发展方式、调整经济结构、加速产业升级，着力统筹城乡发展、保障改善民生、促进社会和谐，实现全市年均经济增长速度高于全省平均水平，保持主要经济指标增幅走在全省前列的良好态势，到“十二五”末，全市生产总值和一般预算收入分别比“十一五”末翻一番，为全面建成小康社会、实现“三个跨越”和开封复兴打下具有决定性意义的基础。

“十二五”时期经济社会发展的主要目标是：

——综合实力明显增强。经济总量大幅度增加，经济发展方式取得重大转变，发展质量和效益明显提高，综合实力再上新的台阶。

——经济结构明显优化。传统产业全面提升，新兴产业加快发展，十大产业集群基本形成，三次产业协调发展，经济结构实现重大调整。

——改革开放明显推进。重要领域和关键环节的改革取得新突破，开放型经济快速发展，承接产业转移取得明显成效，全民创业的社会氛围基本形成。

——城市形象明显改善。老城区改造基本完成，东区、南区建设协调推进；新区建设初具规模，基础设施和综合服务体系基本建成，一批体现新区风貌的重大标志性、功能性项目建成使用。

——农村面貌明显改观。农村基础设施更加完善，综合生产能力持续加强，农村服务业协调发展，现代农业加快推进，新型农民培育成效显著，农村经济全面发展。

——社会建设明显加强。各项社会事业全面发展，社会保障体系更加健全，基本公共服务明显改善，城乡居民收入水平、生活质量和健康水平普遍提高，社会更加和谐稳定。

实现上述发展目标，要把握以下基本原则：一是必须以科学发展为主题。以科学发展为主题，是时代的要求。要把科学发展观的要求体现到经济社会发展的各个方面、各个环节，更加注重以人为本，更加注重全面协调可持续发展。二是必须以加快转变经济发展方式为主线。把加快转变经济发展方式作为推动科学发展的战略任务，贯穿经济社会发展全过程和各领域，坚持在发展中促转变、在转变中谋发展。三是必须以建设全省经济社会发展核心区为战略目标。围绕中原经济区战略目标、战略布局、战略重点，解放思想、融入全局，发挥优势、全面推进，通过五年努力，使开封经济、社会、文化、生态全面发展，核心竞争力不断增强，在加快中原经济区建设和河南振兴中发挥更大作用。四是必须以富民强市为中心任务。坚持富民优先、强市为要，把保障和改善民生作为各项工作的出发点和落脚点，着力构建“一个载体、三个体系”，加快新型工业化、新型城镇化，推进农业现代化，努力实现富民与强市的有机统一。

各位代表!

《开封市国民经济和社会发展第十二个五年规划纲要（草案）》及说明已印发各位代表，请与《政府工作报告》一并审议。

我们坚信，有省委、省政府和市委的正确领导，有全市人民的齐心协力、奋勇拼搏，再大的困难也阻挡不了我们跨越式发展的步伐，我市“十二五”发展目标一定能够如期实现！

三、切实做好2011年工作，努力实现“十二五”良好开局

2011年是“十二五”规划的起步之年，做好今年各项工作，意义重大。2011年经济社会发展主要预期目标是：生产总值增速高于全省平均水平；地方财政一般预算收入增长15%以上；全社会固定资产投资增长22%；社会消费品零售总额增长17%；外贸出口增长21%；实际利用外商直接投资增长25%；居民消费价格涨幅控制在4%左右；人口自然增长率控制在6.5‰以内；新增城镇就业人员6万人以上；城镇居民人均可支配收入实际增长10%；农民人均纯收入实际增长9%；节能减排完成省下目标。

为完成上述目标，要重点抓好以下几个方面的工作：

（一）强力推进工业强市战略，加快向新型工业化城市跨越。坚定不移地实施工业强市战略，加快改造提升传统产业，积极培育战略新兴产业。推进工业结构优化升级。围绕开发精品、拉长链条、项目拉动、技改提升，着力培育产业发展新优势，改造提升装备、化工、食品、纺织服装、生物医药、木业等传统优势产业；坚持龙头带动、基地支撑、承接转移、高端突破，积极培育、加快发展汽车及零部件、新材料、光伏、电子信息等战略新兴产业，尽快形成十大产业集群。培育重点骨干企业。继续实施重点企业和高成长型企业培育工程，进一步完善扶持措施，重点培育壮大奇瑞汽车、晋开集团、开封空分、龙宇化工、东磁集团、福甬纺织、新大新、重装集团等重点工业企业，大力支持高成长型企业发展，力争2011年全市主营业务收入超10亿元的企业突破20家，超20亿元企业达到5家以上。推动企业重组上市。加强对上市后备企业的培育和辅导，推进平原非标、得胜锅炉、依生药业、开药集团等企业加快上市；支持天地药业等企业引入战略投资者和优势资本，推动企业战略重组。狠抓工业项目建设。全力推进129个重大工业项目实施，突出抓好“十大工业提升工程”，力争全年工业投资增长24%，增强工业经济发展后劲。创建新型工业化产业示范基地。围绕我市十大产业集群，以项目带动和产业转移为主要抓手，以打造战略新兴产业专业特色工业园区为平台，力争创建国家级新型工业化产业示范基地1个以上，省级2个以上，进一步优化工业布局。加快推进信息化建设。以“两化融合”为主线，实施两化融合试验区示范工程，提高产业集聚区信息化水平，着力在重点企业和高成长型企业推广信息技术，以信息化助推工业化。

（二）大力发展现代农业，巩固农业农村好形势。坚持把解决“三农”问题作为全部工作的重中之重，大兴水利强基础，狠抓生产保供给，力促增收惠民生，着眼统筹添活力，努力实现农业增效、农民增收、农村发展。加强水利基础设施建设。加大中小河流治理和水利薄弱环节建设力度，完成贾鲁河、铁底河、贺李河等河道年度工程治理任务。加快大中型灌区续建配套与节水改造工程建设步伐，实施赵口灌区引黄入杞工程，突出抓好小型农田水利建设，创新农田水利基本建设长效机制，增强农业防灾减灾能力。提高农产品供给能力。扎实推进粮食生产核心区建设，力争粮食总产量保持在50亿斤以上。深入推进农业结构调整，继续抓好小麦、玉米、花生、瓜菜四个百万亩优势农产品产业带建设。积极推进菊花产业化发展。全面抓好“菜篮子”工程，推动大蒜、辣椒、胡萝卜、食用菌等特色农业加快发展。认真抓好优质畜产品生产加工基地建设和规模养殖场标准化升级改造工作。积极发展农业产业化经营。支持龙大集团、包公食品、河南大用等农业产业化龙头企业发展，增强农副产品加工转化能力，力争培育2―3家产值超10亿元农副产品加工龙头企业。鼓励发展各类农民专业合作经济组织，新扶持30家市级示范社和15家市级农机专业合作社，提高农业组织化程度。拓宽农民增收渠道。加快农民培训基地建设，提高农民职业技能和创收能力，多渠道增加农民收入。大力发展劳务经济，引导农村劳动力向非农产业和城镇有序转移，增加农民工资性收入。深化农村体制改革，积极探索农村土地承包经营权和宅基地使用权的财产化形式，完善土地流转服务体系，增加农民财产性收入。认真落实强农惠农政策。坚持财政支出重点向农业农村倾斜，确保用于农业农村的总量、增量、增幅、比重均有提高。引导金融机构加大涉农信贷投放力度。按照国家统一部署，稳步提高重要粮食品种最低收购价。加大对农业政策性保险的支持力度，增加保费补贴品种，扩大农业补贴规模。扎实推进社会主义新农村建设。以“百村示范”工程为抓手，不断改善农村生产生活条件和村容村貌。继续推进农村基础设施建设，再解决20万人的安全饮水问题，改造农村公路300公里、大中危桥600延米，改造农村危房1万户。实施贫困村整体推进扶贫，完成5万人脱贫。

（三）大力发展文化旅游产业，做大做强第三产业。发挥优势，挖掘内涵，培育精品，主打宋文化品牌，加快发展文化旅游产业，大力发展现代服务业，推动第三产业健康发展。强化文化旅游产业项目谋划和建设。继续深入实施宋都古城风貌保护与重现工程，完成城墙保护四期工程，启动五期工程建设；继续抓好宋都水系、繁塔·禹王台景区开发、大宋文化博物馆·开封城摞城新郑门遗址等一批重大文化旅游项目建设，打造国际文化旅游城市。做好黄河生态旅游区规划，适时启动黄河生态旅游区建设。突出抓好旅游业发展。加强景区建设，优化景区周边环境，提升旅游服务档次和质量。加大文化旅游资源整合力度，着力打造以开封文化旅游投资集团有限公司为龙头、各类文化旅游企业竞相发展的文化旅游产业集群，提升我市文化旅游产业整体竞争力。加快旅游资源整合，深度开发旅游产品，科学编制全市旅游线路，加大宣传推介力度，推动我市旅游业向精品化、国际化发展。加快清明上河园上市步伐。着力提升宋文化影响力。继续抓好中国（开封）菊花花会、中国（开封）清明文化节、中国收藏文化（开封）论坛、大型水上实景演出《大宋·东京梦华》等一批具有较强影响力的宋文化品牌建设，加大汴绣、北宋官瓷、木版年画等一批具有宋文化特色的文化品牌开发挖掘力度，充分发挥宋文化研究院的作用，进一步丰富宋文化内涵，提升开封大宋文化在全国和世界的知名度。大力发展现代服务业。加快香港爪哇大型休闲购物中心、丹尼斯百货、家乐福百货、新玛特购物广场、大润发大型购物商城、苹果园综合市场等商务、旅游、休闲购物服务中心项目建设。发挥饮食文化优势，整合提升餐饮服务业，培育扶植开封餐饮业品牌。继续改造升级农贸市场和夜市。大力发展家庭服务业，重点发展家政服务、社区服务、养老服务。积极发展健身休闲产业。培育扶持生产性服务业发展，突出抓好现代物流业，加快中原农副产品物流园区等现代物流项目建设。鼓励发展金融保险服务业，支持浦发银行等新入汴股份制银行发展，吸引更多的股份制银行在开封设立分支机构。

（四）加快开封新区和产业集聚区建设，打造经济社会发展核心增长极。把开封新区和产业集聚区作为促进经济社会发展全面跨越的重要载体，加强基础设施建设，健全配套服务体系，狠抓项目、企业和人口聚集，加快开封新区和产业集聚区开发建设。强力推进开封新区建设。抢抓开封经济开发区升格为国家级经济技术开发区的机遇，在更广阔的平台上发挥对外开放桥头堡、跨越式发展排头兵的作用，努力实现引资、投资双“倍增”。强力推进基础设施建设，积极运作新区基础设施建设投资公司，开辟多元化融资渠道，加快道路、给排水、供电、供气、供热、污水处理、通信网络等基础设施建设，确保三期、四期基础设施和一批BT项目工程如期建成，完成建设里程35公里。加快黑岗口调蓄水库建设。加快年产8万辆轻卡、30万辆微车及农用机械、工程机械的奇瑞（河南）汽车制造基地项目建设，力争上半年建成投产；抓好总投资29亿元的河南煤化集团大型空分设备、总投资19亿元的河南煤化集团重装基地、总投资15亿元的晋开国际新城、总投资12亿元的金融服务中心等40个重点项目建设；启动北大方正集团综合开发项目建设，加快运粮河组团开发。积极推进职教园区、开封高中西校区、开封市中心医院、解放军第155医院等配套服务设施建设，增强新区吸纳能力。同时，着力引进一批投资强度大、资源消耗低的环境友好型项目，招徕更多的世界500强、国内500强企业投资新区。加快迁村并居步伐，启动横堤铺、后胖，王府寨，斗门迁村并居工程建设；积极推进瞿家砦、野厂迁村并居工程建设；加快闫砦、毛砦迁村并居工程建设进度，力争年内完成建设面积40万平方米。着力加快产业集聚区建设。按照“企业（项目）集中布局，产业集群发展，资源集约利用，功能集合构建，农村人口向城市转移”的总体要求，加快完善政策支持体系，建立健全集聚机制、激励机制和服务机制，积极吸引特色优势产业、各种生产要素和优势资源向集聚区汇聚。加大产业集聚区道路、供排水、标准化厂房等基础设施建设力度，完善公共服务体系，引导学校、医院、住宅、服务网点等配套服务机构加快向产业集聚区延伸。大力承接产业和人口转移，力争全市产业集聚区完成工业投资215亿元，主营业务收入超百亿元的产业集聚区达到3个，把产业集聚区建设成为促进经济发展、加速产城融合的载体和平台。

（五）积极推进城乡建设，加快城镇化进程。坚持把城镇化作为“三化”协调发展的关键环节，全面落实全省城乡建设三年大提升行动计划，以产兴城、以城带乡，加快构建现代城镇体系。抓好老城区开发改造。深入实施宋都古城风貌保护与重现工程，启动水系一期改造，加快水系二期周边开发，加快老城区1号、2号地块和棚户区更新改造，推进老城区3号地块更新改造，积极开发延庆观周边，改造“田”字块周边棚户区。启动鼓楼复建工程。加强对双龙巷等历史街区的保护修缮。强力推进城中村改造。加快城市基础设施建设，启动开封火车站扩建及周边综合改造、郑开城际铁路宋城路站、宋城路公交枢纽站、金明大道公交场站和仁和屯公交场站建设，实施新曹路和魏都路（黄河路—五一路）改造，完成劳动路北延、东外环路北延、电视塔南路和西路、内顺城路（大梁路—向阳路—迎宾路）、孝严寺街（内顺城路—龙亭西路）等道路建设，打通“断头路”，进一步完善城市路网。继续改善城市背街道路、照明等基础设施，搞好大梁路、西门大街、东西大街道路绿化整治。实施东护城河截污清淤及东区污水处理厂配套管网工程建设，完善东区排水体系。统筹谋划东区、南区的发展，高标准制定规划，促进城区协调发展。认真落实国家房地产调控政策，促进房地产市场健康发展。加大交通基础设施建设力度。加快郑汴物流通道开封段、郑民高速开封段、连霍高速开封段拓宽、大广高速公路开封东连接线、郑州至开封城际铁路等一批重点工程建设。启动郑开城际铁路开封段延伸、郑州至徐州客运专线开封段及火车站建设。尽快启动国、省干线道路“二升一”工程，加快市区到各县国、省干线道路升级改造，打造“半小时”交通圈。加快县城和小城镇建设。把县城作为加快城镇化的重点，以产城融合为导向，加强基础设施建设，强化产业支撑，吸引各种要素向县城汇集，不断增强承接中心城市辐射和带动乡村发展的能力。积极推进开封中心城区与开封县城的一体化发展，拓展城市发展空间。把小城镇作为统筹城乡发展的重要节点，着力抓好各乡镇所在地的规划和发展，支持朱仙镇等基础条件较好的中心镇向小城市发展。重点解决好进城务工人员的就业、安居、子女就学、社会保障、户籍管理、承包土地、宅基地和集体财产权益等问题，吸引更多农村人口向城镇转移。加强城市管理。健全城市数字化管理系统相关体制机制，建立精确、敏捷、高效、全时段、全方位覆盖的城市管理新模式；加大城市美化、绿化、亮化、净化力度，突出抓好市容环境、市政设施、园林绿化等方面的精细化管理；加强城市交通综合整治；继续深化创“三城”活动，着力提高市民文明素质和城市形象。积极稳妥推进新型农村社区建设。结合村庄布局规划，积极推进城乡公共资源均衡配置，逐步实现城乡基本公共服务均等化，不断改善农村居民生产生活条件，使城镇化和新农村建设相互促进、协调发展。坚持规划先行、就业为本、群众自愿、量力而行的原则，因地制宜、分类推进新型农村社区建设。将开封新区、各产业集聚区以及城市建成区内的城中村统一纳入城市发展规划，加快城市化步伐；对城乡结合部、县城及中心城镇周边村庄，要加快转变生产方式、就业方式，促进生活方式由农民向市民转变，建设新型农村社区；其他村庄要在不断改善生产生活条件，提高基础设施和公共服务水平的前提下，逐步创造条件建设新型农村社区。

（六）加强科技创新、资源节约和环境保护，转变经济发展方式。把加强科技创新、节能减排和环境保护作为转变发展方式的重要抓手，努力实现节约发展、清洁发展、可持续发展。加快科技进步和创新。全面实施自主创新体系建设和发展规划，出台鼓励创新的政策措施，加快创新型社会建设。围绕人才培养、引进、使用等关键环节，制定人才战略规划，实施重大人才工程，创新人才发展机制。深入实施质量兴市和品牌带动战略，建成国家级空分设备检测中心，力争培育1—2个中国名牌产品企业。继续推进与中国农业大学等高校的合作，争取国家更多重大科研项目和发明专利在开封落户转化。建立节能减排长效机制。严格落实目标责任，健全法规制度，完善目标责任考核评价体系。进一步发挥市场作用，健全激励和约束机制，促使节能减排与企业自身利益相结合，促使企业加快淘汰落后产能、加快技术改造升级和产品结构调整，增强企业和全社会节能减排内生动力。严格投资项目节能评估审查，从源头上控制能耗不合理增长。大力淘汰落后产能，继续实施建筑节能、公共机构节能等八大节能工程。全面推广清洁生产，开展重大节能技术和高效节能产品产业示范。开展绿色建筑和低碳社区示范项目建设，大力发展循环经济。加强环境保护。加强主要污染物排放总量控制，深入推进重点区域、重点流域综合整治和重金属污染治理。控制农村面源污染。认真实施主体功能区规划，切实加强重点生态功能区保护和管理。继续实施水环境生态补偿。完善城镇污水处理厂建设，对已建成的城市污水处理厂进行升级改造和管网配套建设。继续推进林业生态市建设，完成造林面积15.7万亩，实现森林资源“双增”目标。强化资源集约节约利用。实施严格的水资源管理制度，积极推进节水技术改造，加快节水型社会建设。强化土地资源管理，坚持最严格的耕地保护制度和最严格的节约用地制度，大力推进土地综合整治，建立健全土地整治、节约集约用地和监督管理机制，严禁违法用地，控制粗放用地，大幅度提高单位建设用地投资强度、单位土地面积产出率。

（七）深化改革开放，增强经济社会发展动力和活力。以更大的勇气和力度，进一步深化改革，扩大开放，不断增强经济社会发展的动力和活力。继续深化企业改革。深入推进国有企业产权制度改革和破产重组工作。做好开化集团、缝纫机总厂、中州机械厂、第二铸造厂、第二玻璃厂、针织厂、联合制革公司等企业破产或重组的收尾工作。切实加强国有资产监管，健全国有资产管理体制，确保国有资产安全保值增值。鼓励、支持和引导非公有制经济发展，营造各种所有制经济依法平等使用生产要素、公平参与市场竞争、同等受到法律保护的体制环境。深入推进政府自身改革和职能转变。进一步减少审批项目，切实转变政府职能。强化社会管理和公共服务职能，提高公共服务质量和效率。以建立和完善财政预算公开制度为重点，扩大公开范围和层次，着力深化政务公开。建立公共资源交易平台。大力推进社会事业领域改革。深化事业单位人事制度改革。推进公益性文化事业单位改革，全面完成经营性文化事业单位转企改制。深化旅游景区管理体制改革。加快医药卫生体制改革，完善基本医疗保障制度，全面实现医疗救助与新农合、城镇职工和居民医保同步结算。做好公共卫生与基层医疗卫生事业单位绩效工资实施工作。加快推动收入分配、投资评审、资源价格、住房公积金管理体制、环保收费等领域的改革。积极承接产业转移。把承接产业转移与调整经济结构、培育产业集聚结合起来，以开封新区和产业集聚区为主要载体，围绕我市十大产业集群，突出龙头带动、市场带动、配套带动、技术带动，创新招商方式，有针对性地承接国际国内大规模产业转移，争取更多的资金流向开封、更多的企业落户开封，培育一批特色鲜明的产业集群。全面拓宽开放领域。降低准入门槛，强化政策扶持，坚定不移地扩大金融、物流、文化、旅游等服务业对外开放，加大教育、卫生、科技、城市公用事业等社会事业领域开放力度，大力推进城乡建设、现代农业等领域对外开放，通过吸引境内外资金参与这些领域建设，弥补我市发展短板，促进城乡、经济社会协调发展。扩大对外经济贸易技术合作。积极引导企业利用国际国内两个市场、两种资源，开展境外投资，对外承包工程，带动产业转移、设备出口、境外劳务输出。坚持出口和进口并重，完成外贸进出口2.9亿美元。加强外派劳务管理，整顿和规范对外劳务市场秩序，净化外派劳务市场。

（八）切实保障和改善民生，促进社会和谐稳定。坚持把民生工作摆在更加突出的位置，统筹安排好各项民生工程，进一步加大资金投入，继续办好15件为民实事。千方百计扩大就业。以发展促就业，全力以赴加快经济发展，提供更多就业岗位；加大对重点群体的培训力度，增强就业培训的针对性和有效性，提高就业能力；加强人力资源市场建设，畅通就业信息，拓宽就业渠道。认真落实并不断完善就业再就业优惠扶持政策，鼓励企业吸纳更多的劳动力就业，引导有创业愿望和具备创业条件的城乡劳动者创业。重点解决高校毕业生、农村转移劳动力、城镇就业困难人员、退役军人的就业问题。进一步完善社会保障体系。继续扩大养老保险覆盖面，落实基本养老保险关系跨地区转移接续办法。建立健全企业退休人员基本养老金、城乡居民低保、农村五保供养和优抚补助对象正常调整机制。扎实做好新农保试点工作。完善城镇职工、城镇居民医疗保险制度和新农合制度，全面实施医疗保险、失业保险、工伤保险市级统筹。切实解决好失地农民生活保障问题，实现被征地农民社会保障制度全覆盖。加强价格监测、监管，保持价格总体水平基本稳定。大规模实施保障性安居工程。强化政府责任，加强土地、财税、金融政策调节，调动社会各方面力量，加大保障性安居工程建设力度，加快棚户区和农村危房改造，大力发展公共租赁住房。整合各类保障性住房建设资金，开工建设廉租房、公共租赁房、经济适用房等保障性住房1.8万套。加大廉租住房租赁补贴发放力度。大力发展社会事业。始终坚持把教育摆在优先发展的位置，认真实施教育改革发展规划纲要，不断加大投入力度，深化教育教学改革，全面实施素质教育，推动教育事业科学发展。完善农村义务教育经费保障机制，抓好中小学校舍安全工程、城镇义务学校改造和农村学校教师周转宿舍等项目建设。优化农村中小学布局，推进教育均衡发展。制定实施学前教育三年行动计划，有效缓解“入园难”问题。加快发展职业教育。大力支持河南大学、黄河水利职业技术学院等高校发展，办好开封大学和开封文化艺术职业学院。抓好重大文化项目和文化惠民工程建设，加强文艺精品创作，做好文化遗产保护工作。加快城区医疗资源整合，完善城市社区卫生体系。继续实施县级医疗机构、中心乡镇卫生院和标准化村卫生室建设项目。全面加强人口和计划生育工作，积极应对人口老龄化问题。加快社会福利园区建设，大力发展残疾人和养老事业。开展全民健身活动，办好市八运会和2011中国郑开国际马拉松赛。加强和创新社会管理。完善社会管理体系，构建党委领导、政府负责、社会协同、公众参与的社会管理新格局。深入推进社会矛盾化解、社会管理创新和公正廉洁执法建设。健全应急管理体系，积极预防和妥善处置各类突发公共事件；加强和改进信访工作；强化安全生产监管，实施安全河南创建规划年度任务，坚决遏制重特大事故发生；加强食品药品监管，完善食品药品监测检验体系，完成食品药品检测中心项目建设，确保食品药品安全。继续加强民族宗教工作。深入开展“双拥”工作，再创全国“双拥”模范城。继续加强城市社区建设，拓展服务功能。加强基层民主建设，认真做好第七届村委会换届选举工作。全面启动“六五”普法工作。完善社会治安防控体系，加强社会治安综合治理，严密防范、依法打击各种违法犯罪活动，保障人民生命财产安全，确保社会大局和谐稳定。

四、加强政府自身建设

做好今年的工作，要贯彻运用“三具两基一抓手”工作方法，深入开展“两转两提”，以领导方式转变加快经济发展方式转变，促进我市经济社会又好又快跨越式发展。

（一）转变政府职能。深化行政管理体制改革，积极推进政府职能向更加注重强化社会管理和公共服务转变，着力创新公共服务供给体制，提高公共服务质量和效率。深化行政审批制度改革，进一步减少审批事项，减少政府对微观经济活动的干预，最大限度地简政放权。

（二）转变工作作风。在创先争优活动中集中整顿干部工作作风，努力建设一支政治坚定、作风优良、求真务实、开拓进取的干部队伍。深入谋划，精心运作，善于把重大事项、重要工作谋划成具体项目，确保任务落到实处、收到实效。深入开展调查研究，做到问政于民、问需于民、问计于民，使发展思路、工作部署、政策措施更加符合实际、更加符合人民群众愿望。切实改进会风、文风，大力精简会议、文件，坚决反对官僚主义、形式主义。

（三）坚持依法行政。严格执行人大及其常委会的决议、决定，自觉接受人大及其常委会的法律监督和工作监督。自觉接受人民政协的民主监督。广泛听取各民主党派、工商联和无党派人士的意见和建议。重视发挥新闻舆论、网络媒体的监督作用。认真贯彻政府信息公开条例，保障群众知情权，接受群众监督。大力推广电子政务。强化行政执法责任制，推进文明执法。健全以行政首长为重点的行政问责制度，加大责任追究力度。加强制度建设，健全行政决策、执行、咨询、反馈、监督等方面的程序和制度，扎实推进政府工作规范化、标准化、科学化。

（四）加强廉政建设。全面落实党风廉政建设责任制，严格执行反腐倡廉各项规定，加强教育、制度、监督、改革、纠风、惩处并重的惩治和预防腐败体系建设，加大审计监督和行政监察力度，严肃查处各类违纪违法案件，坚决纠正损害群众利益的突出问题和部门行业不正之风。加强政府采购管理，坚决反对铺张浪费，努力建设廉洁政府。

各位代表！

蓝图催人奋进，事业任重道远。让我们紧密团结在以胡锦涛同志为总书记的党中央周围，高举中国特色社会主义伟大旗帜，深入贯彻落实科学发展观，紧紧依靠、团结带领全市人民，以更加振奋的精神、更加开阔的视野、更加务实的作风，努力把“十二五”规划的宏伟蓝图变为美好现实，为开创开封更加美好的明天而努力奋斗！
